# Infrared radiation from hot cones on cool conifers attracts seed-feeding insects

**DOI:** 10.1098/rspb.2008.0742

**Published:** 2008-10-21

**Authors:** Stephen Takács, Hannah Bottomley, Iisak Andreller, Tracy Zaradnik, Joseph Schwarz, Robb Bennett, Ward Strong, Gerhard Gries

**Affiliations:** 1 Department of Biological Sciences, Simon Fraser UniversityBurnaby, British Columbia, Canada V5A 1S6; 2 British Columbia Ministry of Forests and RangeSaanichton, British Columbia, Canada V8M 1W4; 3 British Columbia Ministry of Forests and Range, Kalamalka Forestry CentreVernon, British Columbia, Canada V1B 2C7

**Keywords:** infrared receptor, infrared radiation, *Leptoglossus occidentalis*, foraging cue, conifer cones, seed feeding

## Abstract

Foraging animals use diverse cues to locate resources. Common foraging cues have visual, auditory, olfactory, tactile or gustatory characteristics. Here, we show a foraging herbivore using infrared (IR) radiation from living plants as a host-finding cue. We present data revealing that (i) conifer cones are warmer and emit more near-, mid- and long-range IR radiation than needles, (ii) cone-feeding western conifer seed bugs, *Leptoglossus occidentalis* (Hemiptera: Coreidae), possess IR receptive organs and orient towards experimental IR cues, and (iii) occlusion of the insects' IR receptors impairs IR perception. The conifers' cost of attracting cone-feeding insects may be offset by occasional mast seeding resulting in cone crops too large to be effectively exploited by herbivores.

## 1. Introduction

Efficient foraging by animals entails the use of informative, accurate and easy to assess cues (Dusenbery 1992; Fawcett & Johnstone 2003). Common long-range foraging cues have visual (Briscoe & Chittka 2001; Kelber 2006), auditory (Yack & Hoy 2003) or olfactory (Field *et al.* 2000) characteristics, whereas short-range foraging cues are typically tactile (Schmidt *et al.* 1993) or gustatory (Hallem *et al.* 2006). Infrared (IR) radiation from endothermic (warm-blooded) vertebrates is a rare-foraging cue used by certain snakes (Grace *et al.* 2001; Moiseenkova *et al.* 2003) and vampire bats (*Desmodus rotundus*; Kuerten & Schmidt 1982, 1985) to locate prey. Few insects are known or speculated to exploit heat or IR foraging cues. The blood-feeding kissing bug, *Triatoma infestans*, appears to possess thermoreceptors that enable it to perceive radiant heat from endothermic prey and estimate its temperature (Lazzari & Núñez 1989). The blood-feeding bug *Rhodnius prolixus* approaches a thermal source guided solely by its IR radiation ([Bibr bib27][Bibr bib28]). The pyrophilic (fire-loving) jewel beetle, *Melanophila acuminata*, Australian flat bug, *Aradus albicornis* and Australian fire beetle, *Merimna atrata*, possess IR receptors on the prothorax or abdomen, and sense forest fires from afar, moving in quickly to lay their eggs in the smouldering bark of burnt trees where their offspring develop virtually free of competition (Evans 1964; Schmitz *et al.* 1997, [Bibr bib27][Bibr bib28], 2008). However, the use of IR radiation from living plants as a long-range foraging cue for herbivores is unknown.

Self-heating of live flowers, inflorescences or cones has been reported in several families of gymnosperm and angiosperm plants, including cycads, arum lilies, Dutchman's pipes, palms, custard apples, winter's bark and magnolias (Thien *et al.* 2000). Thermogenicity in these primitive seed plants is usually thought to enhance the production and dissemination of floral scents rendering the plants more attractive to beetle, bee or fly pollinators (Seymour & Schultze-Motel 1997). Cycads use an intriguing heat- and odour-mediated strategy to direct the movement of insect pollinators between their cone-like male and female flowers (Terry *et al.* 2007). Self-heating of plant tissue may also reward pollinators by keeping them warm in floral chambers where they remain overnight (Meeuse & Raskin 1988).

The thermal gradient between warm plant tissue and its external environment is steep (Baierlein 1999), making conductive heat perceptible over only a very short range. Thus, conductive or convective heat is not a suitable long-range foraging cue to pollinators or herbivores, but may reward ectothermic pollinators at close range (Kevan 1975; Seymour *et al.* 2003; Dyer *et al.* 2006). IR radiation from warm plant tissue, by contrast, is perceptible from afar (Baierlein 1999). We expected significantly greater IR radiant exitance (=total radiative flux leaving a surface) from conifer cones than needles because the surface area to mass ratio of cones is conducive to absorbing solar radiation and heating above ambient temperature. The cone surface also tends to reflect effectively solar mid- and long-range IR radiation. Finally, cones may be thermogenic because they are a nutrient sink and a site of high metabolic activity during seed development. Here, we show that the western conifer seed bug, *Leptoglossus occidentalis* Heidemann (Hemiptera: Coreidae), a tissue specialist herbivore that forages during the photophase and feeds on the contents of seeds within the cones of many conifers (Blatt & Borden 1999; Strong *et al.* 2001), uses IR radiation from developing cones as a long-range foraging cue. We present data revealing that (i) cones are warmer and continuously emit more near-, mid- and long-range IR radiation than needles, (ii) seed bugs possess IR receptive organs and orient towards experimental IR cues, and (iii) occlusion of the insects' IR receptors impairs IR perception.

## 2. Material and methods

### (a) Experimental insects

Adult *L. occidentalis* were collected in late August 2006 from the Sechelt Seed Orchards (Canadian Forest Products, Ltd.), Sechelt, British Columbia, Canada (123°43′ W, 49°27′ N). Males and females were separated based on the presence of a marked abdominal angularity (male) or ovipositor (female) and were housed in separate mesh cages (30×30×50 cm). Cages were kept outdoors under ambient temperature and light conditions; they contained potted grafts or seedlings of western white pine, *Pinus monticola*, or of mugo pine, *Pinus mugo*. Insects were provisioned ad libitum with water, fresh pine cones (when available) and seeds of western white pine or Douglas-fir, *Pseudotsuga menziesii* var. *menziesii*.

### (b) Temperature measurements and thermographs of *in situ* cones and needles

On each of five separate dates (29 May, 19 June, 17 July, 15 August, 23 September 2006), cones and clusters of needles were randomly selected for thermographing and photographing with an AGEMA Thermovision 550 camera (FLIR Systems Ltd., Burlington, Ontario L7L 5K2, Canada) sensitive in the 3–5 μm IR range, and a Nikon Coolpix 995 (Nikon Canada, 5-13511 Crestwood Place, Richmond, BC, V6V 2E9). The recording distance was kept at 2 or 10 m, and the emissivity (the ratio of the radiation emitted by a surface to the radiation emitted by a black body at the same temperature) at 0.75 (Ohman 2006), whereas other parameters (atmospheric and reflected apparent temperature, relative humidity) varied according to the weather conditions. On 19 June 2006 at 14.00–15.00, area and spot-apparent temperatures recorded thermographically were compared with concurrent direct temperature measurements with a thermocouple (Omega HH 506 RA multilogger with type K 0.032 thermocouples; Stanford, CT 06907-0047) inserted under a scale of an *in situ* cone or into a nearby needle exposed to direct sunlight. Additional thermographs were taken on 20 May 2008 with a Fluke TI-20 thermal imager (Fluke Corp. Everett, WA 98206, USA) sensitive in the 8–20 μm IR range, following the procedures outlined above.

During the April to August 2007 growing season, cones on five Douglas-fir and six western white pine trees in the Sechelt Seed Orchards were subjected every two weeks to thermographic, photographic and IR-filtered imaging (Hoya R72 Infrared, 49 mm filter, THK Products, Inc., 2360 Mira Mar Avenue, Long Beach, CA 90815). The same type of imaging was applied to cones of select lodgepole pine, *Pinus contorta* var. *latifolia*, Douglas-fir, western white pine, Engelmann spruce, *Picea engelmannii* and western larch, *Larix occidentalis*, trees in the Kalamalka Seed Orchard (BC Ministry of Forests and Range), Vernon, British Columbia (BC), Canada (119°16′ W, 50°14′ N).

### (c) Spectrometric profiles of cones and needles

In late summer, cones and clusters of needles were randomly selected to determine their spectrometric profiles in the ultraviolet through near-IR range under natural sun or halogen light. Measurements employed both an HR4000 high-resolution spectrometer (responsive: 0.200–1.100 μm; greater than 50% sensitivity: 0.300–0.750 μm; Ocean Optics, 830 Douglas Avenue, Dunedin, FL 34698) and an NIR256-2.5 extended-range near-IR spectrometer (responsive: 0.900–2.550 μm; greater than 50% sensitivity: 1.500–2.550 μm; Ocean Optics). On 10 September 2007 between 13.00 and 15.30, readings of absorbance and reflectance were taken of three Douglas-fir cones, three white pine cones and three samples each of closely packed needles of Douglas-fir and white pine. The measurement distance was 2 cm, the atmospheric temperature 18°C and the relative humidity 40 per cent.

### (d) Response of seed bugs to IR radiation cues in laboratory experiments

**Figure 1:**
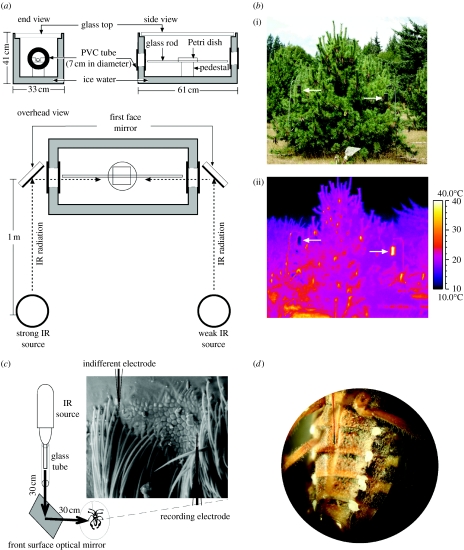
(*a*) Schematic of the experimental design for testing response of western conifer seed bugs to IR radiation. Strong or weak IR radiation was emitted from a Pyrex glass flask (1000 ml) containing heated (42°C) or ice-cooled (4°C) water. Drawing is not to scale. (*b*(i)) Photograph and (ii) thermograph of paired, adhesive-coated PVC pipe traps (marked by arrows) that were left empty, absorbed solar energy, heated up and emitted strong IR radiation (right trap) or were filled with ice water, remained cool and emitted weak IR radiation (left trap). The temperature bar to the right of the lower image reveals that the empty trap and cones had about the same temperature. Images were taken on 15 August 2006 at 13.44 (distance=10 m, temperature=23°C, reflected apparent temperature=22.4°C, R.H.=79%) in a seed orchard near Sechelt, British Columbia, Canada. (*c*) Schematic of the set-up for electrophysiological recordings of IR receptor potentials in response to IR radiation. Arrows represent path of IR radiation. Drawing is not to scale. (*d*) Photograph of the ventral surface of a seed bug abdomen with occluded IR receptors. The specimen shows droplets of an IR opaque suspension of silica gel and acrylic paint on each of its eight IR receptor sites.

Attraction responses of male and female seed bugs to IR radiation were tested in a cooled chamber designed to eliminate external thermal cues (figure 1*a*). The chamber consisted of a glass aquarium (50.5×26.7×33 cm high) nested inside a larger aquarium (61×33×41 cm high) with ice water between them, and a glass top covering the inner aquarium (design modified from [Bibr bib27][Bibr bib28]). A PVC tube (7 cm i.d.) was sealed between the two aquaria in each of the two end sections to exclude water, allowing IR radiation to enter the inner aquarium. The air temperature (10±1°C) near both openings within the inner aquarium and the water temperature (4.0±0.5°C) were monitored continuously with type K 0.032 thermocouples.

Insects were released from an etched open Petri dish (10 cm i.d.) resting on a pedestal (9×9×10 cm) within the inner aquarium (figure 1*a*). They were exposed to IR radiation entering the chamber through the PVC tubes, and responded by climbing onto and walking towards the distal end of an etched glass rod (0.8×45 cm) inserted through the Petri dish.

Strong and weak IR radiation was generated from Pyrex glass flasks (1000 ml) containing heated (42°C) or ice-cooled (4°C) water (figure 1*a*). These temperatures are biologically relevant because seed bugs in early spring seed orchards may experience atmospheric temperatures between 2 and 12°C, with cone temperatures as high as 50°C on sunny days. Front surface optical mirrors (13×13 cm; SEA-UV protected, aluminium broadband, SiO_2-_coated; Praezisions Glas & Optik GmbH, D-58640 Iserlohn, Germany) reflected IR radiation (but not conductive or convective heat) into the inner aquarium. To ensure that seed bugs could perceive IR radiation stimuli while they were residing in the Petri dish or walking on the glass rod, a horizontal laser was used to position properly both IR sources and mirrors. A thermographic camera (AGEMA Thermovision 550), sensitive in the 3–5 μm wavelength range and able to resolve temperature differences of 0.1°C, confirmed the *apparent* temperature of, respectively, 32±2°C and 6±2°C that was reflected by the mirrors.

At the beginning of the photophase, single seed bugs were transferred from outdoor cages to a Petri dish and allowed to acclimatize for 1–4 hours at room temperature in darkness. For each experimental replicate, the strong IR source was randomly assigned to one end of the aquarium, the weak IR source to the other. A single seed bug was then placed into the Petri dish within the inner aquarium. Individuals that mounted the rod and walked to one end within 15 min were classed as responders and were included in statistical analyses (*Χ*^2^-test with Yate's correction for continuity (Zar 1984), *α*=0.05). All others were classed as non-responders.

Between replicates (*n*=45 in experiments 1 and 2), the glass Petri dish and rod were washed and baked overnight at 120°C to remove potential semiochemical cues. Interior surfaces of the chamber were wiped with a paper towel saturated with a Sparkleen solution after every 12 replicates and aired for more than 12 hours.

In experiments 4 and 5, IR receptors were occluded (figure 1*d*) by applying an IR-opaque suspension of silica gel and acrylic paint (1.33 g and 59 ml; Craft Smart White, Plaid enterprises Inc., Norcross, GA 30091-7600) with a single-bristle paintbrush on each of the eight candidate IR receptor sites. Control insects received an equivalent amount of the suspension lateral to each receptor site. All such treated insects were kept isolated with food and water for 24–48 hours to allow the suspension to dry. Behavioural responses to IR radiation sources were tested and recorded as described above. Data including those of non-responding (NR) insects were analysed with *g*-test rather than *Χ*^2^-test because the minimum requirement of five observations per category was not always met.

### (e) Response of seed bugs to IR cues in a field experiment

At the lower part of Sechelt Seed Orchards (see above), 10 white pine trees bearing 40–80 cones each were selected, with at least 10 m between trees. An experimental trap was placed to the east and west side of each selected tree. Traps were made of a black PVC pipe with a permanent cap at the bottom and a white removable lid, suspended from an L-shaped pole by thin wire approximately 2 m above ground (figure 1*b*). The traps were coated with adhesive Tangle-Trap insect trap coating (The Tanglefoot Company, Grand Rapids, MI 49504-6485) to retain seed bugs that landed on them. One randomly assigned trap per tree was filled with ice water (weak IR radiation), the other left empty to absorb solar radiation and heat up (strong IR radiation) (figure 1*b*).

Between sunrise and sunset on 11–22 August 2006, traps were checked every 30 min, and ice was added to ‘ice water traps’ every 120–180 min. Seed bugs present on traps were counted and removed. Data were analysed with Student's *t*-test (Zar 1984).

### (f) Environmental scanning electron microscopy of live seed bugs

Environmental scanning electron micrographs (ESEM) of live seed bugs were recorded by an FEI Quanta FEG 400 instrument (Shottky-type field-emission source; accelerating voltages: 0.2–30 keV; resolution: 3.5 nm at 3 kV in low-vacuum mode) at the Pulp and Paper Institute of Canada (Paprican). ESEMs are images of biological samples obtained without coating them. By introducing water vapour into the chamber, the pressure is raised and the moisture adsorbs on the surface of the sample, acting as the charge dissipater, thus enabling imaging of uncoated biological samples (Donald 2003).

### (g) Innervation of IR receptor sites

To visualize the nervous system, it was stained with intravital methylene blue (Mahoney 1973). A 1.0–2.5 ml solution of reduced methylene blue was injected into the dorsal metathorax of live specimen's restrained dorsal side up. After 30 min, the dorsal sclerites from the meso- and metathorax, and from all abdominal segments, were carefully removed. The alimentary tract, portions of the tracheal system and reproductive organs were then excised to expose the central nerve cord. After careful removal of fat, trachea and muscle tissues, individual nerve fibres could be traced from the ventral nerve cord to their terminus.

### (h) Electrophysiological recordings

Electrophysiological recordings from hypothesized IR receptor sites were conducted with glass capillary electrodes (1.1 mm i.d. pulled to approx. 10 μm o.d.) filled with a 0.1 M KCl solution. The insect with its ventral side up was restrained with utility wax (Kerr, Romulus, MI 48174) on a brass platform. The reference and recording electrodes were micro-manipulated (Leitz micromanipulator M, Vienna, Austria) into the perimeter of IR receptor sites (figure 1*c*). Chlorinated silver wires were used to maintain electrical contact between the electrodes and the input of the preamplifier. DC-coupled analogue signals were detected through a probe (INR-II, Syntech_R, The Netherlands), captured and processed with data acquisition controller (IDAC signal interface box, Syntech_R, The Netherlands), and analysed with software (EAG v. 2.4, Syntech_R, The Netherlands) on a personal computer. A beam of IR radiation (but not conductive or convective heat) from a glass tube (5 mm i.d.) encasing a heated soldering iron was directed via a front surface optical mirror (13×13 cm) onto the IR receptor. The apparent reflected spot temperature was 50±10°C (figure 1*c*). Receptors not being recorded were draped with IR-opaque tape to reduce cross reactions. A programmable, custom-built electronic camera shutter (R. Holland, Science Technical Centre, Simon Fraser University), positioned between the mirror and IR source, continuously intercepted the IR beam, except for intermittent 3 s intervals during which IR receptors were exposed to IR radiation.

## 3. Results and discussion

**Figure 2:**
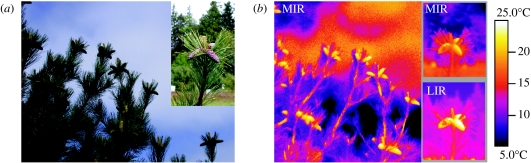
Conspicuousness of western white pine cones in mid-range (3–5 μm; MIR) and long-range (8–20 μm; LIR) IR spectrum thermographs. (*a*) The visual spectrum photographs and (*b*) the thermographs were taken on 15 May 2008 at 13.15 (distance=10 m, temperature=10°C, reflected apparent temperature=19°C, R.H.=53%) in a seed orchard near Sechelt, British Columbia, Canada. Insets show images of cones taken from 2 m. The temperature bar to the right of the paired images reveals that cones are up to 15°C warmer than foliage under high-cloud conditions.

**Figure 3:**
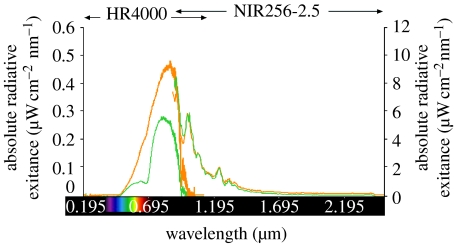
Representative measurements of absolute radiant exitance (ultraviolet through near-IR (0.2–2.5 μm) wavelengths) from needles (green) and cones (orange) of a western white pine branch under halogen light. Two separate instruments (see text for details) were used to take measurements.

**Figure 4:**
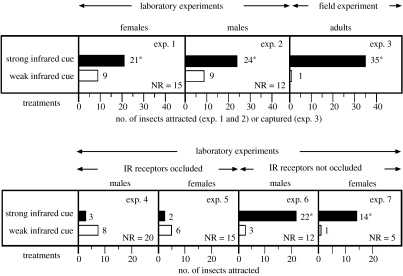
Attraction of western conifer seed bugs to IR radiation in two-choice experiments (figure 1*a*,*b*). In experiment 3, the mean number (±s.e.) of insects captured in 10 replicates was 3.5 (±0.72) and 0.1 (±0.1) in treatment and control stimuli, respectively. In each experiment, an asterisk indicates a significant preference for a particular stimulus; *Χ*^2^-test with Yate's correction for continuity, *p*<0.05 (experiments 1 and 2), Student's *t*-test, *p*=0.0003 (experiment 3) and *g*-test, *p*<0.05 (experiments 4–7). Numbers beside bars indicate the number of insects responding to test stimuli. NR insects are included in the *g*-test statistical calculation.

In surface temperature measurements and thermographs of cones and needles throughout the day, cones were found to be up to 15°C warmer than needles and to emit significantly stronger mid-range (3–5 μm) and long-range (8–20 μm) IR radiation (figure 2). These differences were recorded throughout spring, summer and autumn for developing cones of western white pine, lodgepole pine, Douglas-fir and Engelmann spruce. Near IR (1.0–2.5 μm) spectra from cones and needles were similar but their visual and very near IR spectra (0.3–1.0 μm) differed (figure 3). In two-choice laboratory experiments, both male and female seed bugs preferred stronger IR radiation over weaker IR radiation (*Χ*^2^-test with Yate's correction for continuity: *p*<0.05; figure 4: experiments 1 and 2). The potential for orientation to a conductive heat source was eliminated by reflecting the IR radiation by 90° with a mirror (figure 1*a*). In a two-choice field experiment with 10 replicates, 35 out of the 36 captured seed bugs were retained on adhesive-coated pipe traps emitting the stronger IR stimulus than on adhesive-coated cold traps with which they were paired (figure 1*b*) (Student's *t*-test, *p*=0.0003; figure 4: experiment 3). That these differential captures of seed bugs on paired traps were also modulated by potential subtle differences in the volatile emission and tackiness of the adhesive is highly unlikely because numerous insects other than seed bugs were retained on all traps.

**Figure 5:**
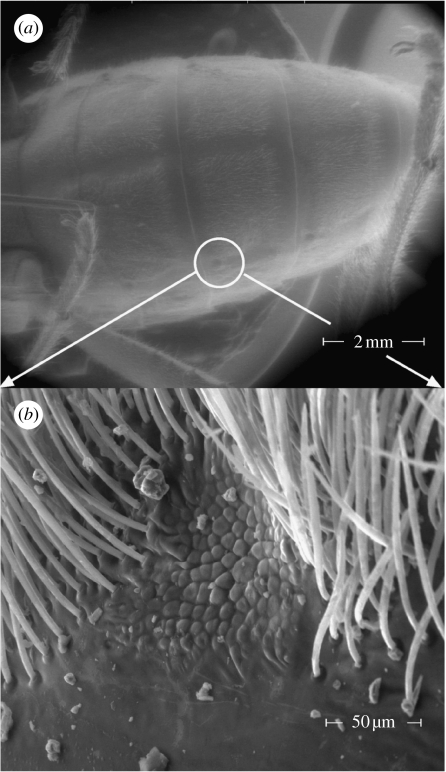
ESEM of IR receptor sites on an adult female western conifer seed bug. The images show receptor locations (marked by circle) on (*a*) the ventral abdomen and (*b*) a close-up of one IR receptor site. IR receptors of males are similar in location, appearance and size.

**Figure 6:**
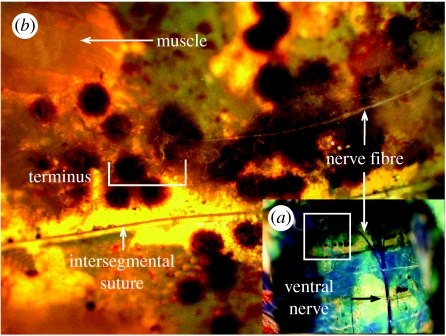
Photographs of the stained nervous system of a western conifer seed bug depicting (*a*) the ventral nerve cord and (*b*) the neuron and its terminus associated with the IR receptor.

**Figure 7:**
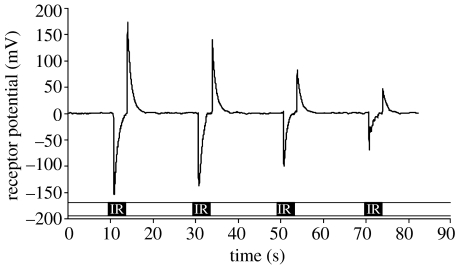
Representative IR receptor potentials in response to IR radiation. The recording was obtained from the left receptor site on the third abdominal segment of a female western conifer seed bug exposed to reflected IR radiation for 3 s periods, as indicated by black bars.

Scanning electron micrographs of the seed bugs' body surface revealed a hypothesized pair of IR receptor sites on each abdominal segment 1–4 (figure 5). Each site has a surface texture distinctively different from that of the surrounding integument and is bordered by setae. Dissection and intravital staining of the nervous system revealed the innervation of the hypothesized IR receptor sites (figure 6). IR receptors of the Australian fire beetle, *M. atrata,* are similar in surface texture and are also located ventro-laterally on the abdomen ([Bibr bib27][Bibr bib28]). Inserting electrodes into the perimeter of a receptor site (figure 1*c*), and exposing that site to intermittent IR radiation, elicited changes in receptor potentials of above 100 mV (figure 7). Consistent responses were obtained particularly from sites located on the second and third abdominal segment. Control recordings from adjacent abdominal segments devoid of IR receptors did not elicit electrical potentials.

The vital role of these receptors in the perception of IR radiation became apparent when they were occluded by silica paint (figure 1*d*), which is impermeable to IR radiation. Seed bugs with occluded receptors were incapable of responding to the stronger IR cue in two-choice experiments (*g*-test, *p*>0.05; figure 4: experiments 4 and 5). Conversely, control insects that were silica-painted near, but not over, IR receptor sites were strongly attracted to the stronger IR cue (*g*-test, *p*<0.05; figure 4: experiments 6 and 7).

We conclude that developing cones emit and reflect IR radiation that seed bugs exploit as foraging cues. In a complex landscape, these IR cues make cones highly apparent (figure 2) to a seed-foraging insect, mediating long- and short-range orientation towards them. Warm cones contrast well against surrounding cool foliage and sky. Unlike visual spectrum cues, emitted IR radiation remains an effective cue at dawn and dusk when thermal contrast against a background is greatest (Baierlein 1999).

IR radiation from host plant tissues may also be easiest to assess. Its perception may require the same receptor(s) irrespective of the trees from which it originates. Visual reflectance spectra (0.30–0.68 μm) of bark (Campbell & Borden 2005) and needles (figure 3) of conifer species reveal little distinction, and even though their volatile chemicals may differ in ratio, there is virtually complete overlap in volatile chemicals represented (Pureswaran *et al.* 2004). For polyphagous conifer seed bugs, perception and integration of IR radiation as a long-range foraging cue might be most ‘economic’.

The observed response of seed bugs in our field experiment to IR radiation is astonishing considering that the plastic pipe traps (figure 1*b*) did not resemble natural cones in form or colour, lacked any olfactory cues, and in the seed orchard competed with the many natural cones.

IR radiation may also be a significant foraging cue for other seed-feeding insects, including species of pyralid and tortricid cone moths and cecidomyiid midges in the genera *Contarinia* and *Mayetiola*. Western boxelder bugs, *Boisea rubrolineata,* which feed on maturing seeds of their boxelder host trees, also responded in laboratory experiments to IR cues (data not shown).

Flowers advertise with fragrance and colour to lure pollen- and nectar-foraging insects, and some even provide ultraviolet guides to their pollen and nectar sources (Kevan *et al.* 2001; Schoonhoven *et al.* 2005). We predict that thermogenic flowers of basal angiosperms advertise, in part, by IR radiation and that their specific pollinators have well-developed IR perception. We further predict that some pollinators of higher angiosperms may use contrasting IR radiation from filled and empty floral nectaries to help them determine profitable inflorescences to visit.

If IR radiation is as outstanding a cone-foraging cue as it appears, one might expect measures that compensate for seed herbivory. The cost of attracting seed-feeding insects may be offset by occasional geographically isolated synchronous production of unusually large seed crops (Henson 1966; Turgeon *et al.* 1994) that cannot be effectively exploited by insect herbivores.

Our findings provide impetus to investigate IR perception as a potentially widespread foraging cue in tissue-specific herbivores, including frugivorous insects and the invertebrate and vertebrate pollinators.
